# Exploration of the prognostic value of the resection of adult brainstem high-grade glioma based on competing risk model, propensity score matching, and conditional survival rate

**DOI:** 10.1007/s10072-022-06557-z

**Published:** 2023-01-06

**Authors:** Dainan Zhang, Haiming Li, Wang Jia

**Affiliations:** 1 Department of Neurosurgery, Beijing Tiantan Hospital, Capital Medical University, Beijing, 100070 China; 2 Beijing Neurosurgical Institute, Capital Medical University, Beijing, China; 3 Henan Provincial Key Laboratory of Neurorestoratology, The First Affiliated Hospital of Xinxiang Medical University, Wei Hui, China

**Keywords:** Resection, High-grade glioma, Brainstem, Competing risk model

## Abstract

**Background:**

Adult brainstem high-grade glioma (HGG) is a refractory disease, and the treatment strategy of resection is still controversial.

**Objective:**

To investigate the prognostic value of brainstem HGG resection in adults.

**Methods:**

We collected 126,386 samples from the Surveillance, Epidemiology, and End Results (SEER) database between 1998 and 2016, and screened 973 patients diagnosed with adult brainstem HGG, who were in turn, grouped into 899 cases of non-resections and 74 cases of resections. Competing risk models were used to screen independent prognostic factors. Propensity score matching (PSM) was performed to reduce the influence of confounding factors. Conditional survival (CS) rate was considered to evaluate the changes in overall survival (OS) and disease-specific survival (DSS) of patients with HGG over time.

**Results:**

Based on the competing risk model and PSM, univariate analysis showed that age ≥ 45 years and male gender were poor prognostic factors for adult brainstem HGG. No previous history of glioma was a beneficial factor. Multivariate analysis revealed only the absence of a history of glioma to be a favorable prognostic factor. Considering the CS rate of the resection group, after the patient had survived for 3 years, the OS and DSS remained unchanged at 100% during the fourth and fifth years, whereas in the non-resection group, the OS and DSS of the patients were 82% and 74%, respectively.

**Conclusion:**

Adult brainstem HGG resection has a poor prognosis in the early stage; however, patients have a potentially significant survival benefit after 3 years of survival.

**Supplementary Information:**

The online version contains supplementary material available at 10.1007/s10072-022-06557-z.

## Introduction

Brainstem high-grade glioma (HGG), a rare and aggressive tumor, occurs mostly in children [1, 2] and rarely in adults, accounting for 1–2% of all adult cases of central nervous system tumors [3–5]. There are crucial nerve centers in the brainstem, such as the cardiovascular movement, respiratory, and swallowing centers. Due to its special anatomical location, surgical resection of brainstem HGG is difficult, making the treatment of brainstem HGG challenging [6, 7]. Studies have reported that the median survival rate of patients that underwent surgical intervention was only 11 months, and the overall survival (OS) rate was very low [8]. Moreover, the patient’s age, gender, radiotherapy, chemotherapy, tumor size, and scope of surgical resection have been reported to be related to HGG prognosis [9–12]. However, OS in adult patients with HGG and related survival factors are still controversial.

Competing events of non-cancer death, such as heart disease, cerebrovascular disease, and influenza, do exist in patients with cancer [9]. Therefore, compared with the Cox survival analysis, the use of a competing risk model would facilitate accurate assessment of the association of predictor variables with outcome events. Moreover, confounding factors are often ubiquitous in observational research. The current study aimed to achieve “randomization” through propensity score matching (PSM) to control the influence of confounding factors on research conclusions. Additionally, we found the conditional survival rate (CS) of patients to change over time. CS can quantify the degree of improvement in the prognosis of patients over time, facilitating the adjustment of long-term follow-up strategies.

Exploration of the prognostic value of expanded resection of adult brainstem HGG based on a single medical center often leads to a small sample size and low statistical power [13–15]. The patient selection bias based on hospital-centered data is often greater than that based on the Surveillance, Epidemiology, and End Results (SEER) data. With the above considerations, the current study aimed to include adult patients with brainstem HGG through the SEER database, based on the competitive risk model, PSM, and CS, to study the prognostic value of resection of adult brainstem HGG.

## Methods

### Baseline data

We collected 126,386 samples from the SEER database. The criteria for inclusion and exclusion were as follows: the histological code of the International Classification of Diseases for Oncology Third Edition (ICD-O-3) was 9380/9401/9440/9441/9442/9451, the age was ≥18 years old, the tumor occurred in the brainstem, and 973 people finally met the criteria. The main variables in this study are non-resection (including no operation/biopsy) and resection (including subtotal resection/total resection). The included covariates were age, gender, race, marital status, diagnosis time, radiotherapy/chemotherapy, tumor size, etc. (Table 1). The SEER database is the authoritative and public source of information about cancer incidence, mortality, and survival rates in the USA. We used SEER-Stat software (version 8.3.5) to download patients’ clinical data. The data are publicly available and do not involve privacy of patients; therefore, so no ethical review was required.

**Table 1:** Sample baseline data

Variables	Total (*n* = 973)	Non-resection (*n* = 899)	Resection (*n* = 74)	*P*
Survival, months median (Q1, Q3)	16 (6, 52)	17 (6, 54)	12 (7, 25.5)	0.15
Outcome, *n* (%)				0.007
Death	629 (64.6)	570 (63.4)	59 (79.7)	
Live	344 (35.4)	329 (36.6)	15 (20.3)	
Outcome 3, *n* (%)				0.01
Death from glioma	500 (51.4)	450 (50.1)	50 (67.6)	
Death from others	129 (13.3)	120 (13.3)	9 (12.2)	
Live	344 (35.4)	329 (36.6)	15 (20.3)	
Age, median (Q1, Q3)	45 (32, 58)	45 (32, 58)	43 (30.25, 56.75)	0.375
Sex, *n* (%)				0.703
Female	448 (46)	416 (46.3)	32 (43.2)	
Male	525 (54)	483 (53.7)	42 (56.8)	
Race, *n* (%)				0.439
Black	104 (10.7)	94 (10.5)	10 (13.5)	
Others	102 (10.5)	92 (10.2)	10 (13.5)	
White	767 (78.8)	713 (79.3)	54 (73)	
Marital, *n* (%)				0.262
Divorced/separated	89 (9.1)	84 (9.3)	5 (6.8)	
Married	505 (51.9)	460 (51.2)	45 (60.8)	
Single/unmarried	291 (29.9)	270 (30)	21 (28.4)	
Widowed/others	88 (9)	85 (9.5)	3 (4.1)	
Diagnosis, *n* (%)				0.95
1998–2004	231 (23.7)	213 (23.7)	18 (24.3)	
2005–2009	255 (26.2)	234 (26)	21 (28.4)	
2010–2012	184 (18.9)	170 (18.9)	14 (18.9)	
2013–2016	303 (31.1)	282 (31.4)	21 (28.4)	
Past history type, *n* (%)				<0.001
GBM	180 (18.5)	142 (15.8)	38 (51.4)	
Others	793 (81.5)	757 (84.2)	36 (48.6)	
Radiotherapy, *n* (%)				<0.001
No	864 (88.8)	845 (94)	19 (25.7)	
Yes	109 (11.2)	54 (6)	55 (74.3)	
Chemotherapy, *n* (%)				<0.001
No	614 (63.1)	583 (64.8)	31 (41.9)	
Yes	359 (36.9)	316 (35.2)	43 (58.1)	
Tumor size, *n* (%)				0.014
Size < 20 mm	151 (15.5)	147 (16.4)	4 (5.4)	
Size ≥ 20 mm	310 (31.9)	278 (30.9)	32 (43.2)	
Unknown	512 (52.6)	474 (52.7)	38 (51.4)	
Age, *n* (%)				0.696
Age < 45	485 (49.8)	446 (49.6)	39 (52.7)	
Age ≥ 45	488 (50.2)	453 (50.4)	35 (47.3)	
Surgery 2, *n* (%)				<0.001
Biopsy	63 (6.5)	63 (7)	0 (0)	
Gross total	22 (2.3)	0 (0)	22 (29.7)	
None	836 (85.9)	836 (93)	0 (0)	
Subtotal	52 (5.3)	0 (0)	52 (70.3)	

Outcome: binary variables (0: live; 1: death); outcome 3: three categorical variables (0: live; 1: death from glioma; 2: death from others); surgery 2: four categorical variables (0: none; 1: biopsy; 2: subtotal resection; 3: gross total resection)

### Competing risk model

In this study, we chose death from brainstem HGG as the end event while deaths caused by other factors were regarded as competing risk events. Cumulative incidence function (CIF) was used to estimate the cumulative occurrence probability of an outcome event, which was then used to process the survival data from multiple endpoints and competing risk events. The CIF for death due to brainstem HGG and other competing risk events was calculated and was grouped by age, race, chemotherapy, etc. Using cmprsk’s R package, we drew a CIF curve for each variable, and performed Gray’s test to identify the difference between brainstem HGG and non-brain stem HGG deaths in CIF. Subsequently, for multivariate competing risk survival analysis, we constructed the Fine-Gray proportional sub-distribution hazards model, and used cmprsk and risk regression to predict the potential risk factors of death from brainstem HGG and death from non-brain stem HGG events.

### Propensity score matching

This study used PSM to balance the clinical data between the resection group and the non-resection group in the SEER cohort, including the following baseline characteristics: age, gender, race, marital status, time of diagnosis, chemotherapy, tumor size, and past history, to achieve the effect of retrospective randomization. First, we used the multiple logistic regression model to calculate the propensity score (PS) of each patient according to surgery type (resection and non-resection). Second, we used the MatchIt package in R software to analyze the data, set the caliper value to 0.02, and evaluated the effect according to the standardized mean difference (SMD) and *P* value.

### Conditional survival rate

Conditional survival rate was estimated from clinical data according to the Berkson-Gage method, and its variance and confidence interval were derived according to the binomial distribution theory. This study mainly analyzed the binary variables of resection and non-resection, the overall survival rate (OS), and disease-specific survival rate (DSS) after 0, 1, 2, 3, 4, and 5 years following surgery. The probability that a patient who has survived *x* years after the initial treatment will survive for another *n* years was expressed as CS(OS/DSS)(*n*) = [(OS/DSS)(*x* + *n*)]/[(OS/DSS)(*x*)].

## Results

### Patient baseline information

In this study, 973 eligible patients were divided into non-resection (*n* = 899) and resection (*n* = 74) groups (Table 1). Including 448 males (46%) and 525 females (54%), the median survival time of non-resection group was 17 months and that of resection group was 12 months; 629 people died (64.6%) and 344 people survived (35.4%) during the follow-up; 570 people (63.4%) died and 329 people (36.6%) survived the non-resection; 59 people (79.7%) died and 15 people survived (20.3%) the resection. However, of the 899 people who underwent non-resection, 450 (50.1%) died due to glioma, whereas 120 (13.3%) did not die from glioma; similarly, 74 underwent resection, 50 (67.6%) died due to glioma, and 9 (12.2%) died from other events.

### KM curve of patient OS and DSS

**Fig. 1:**
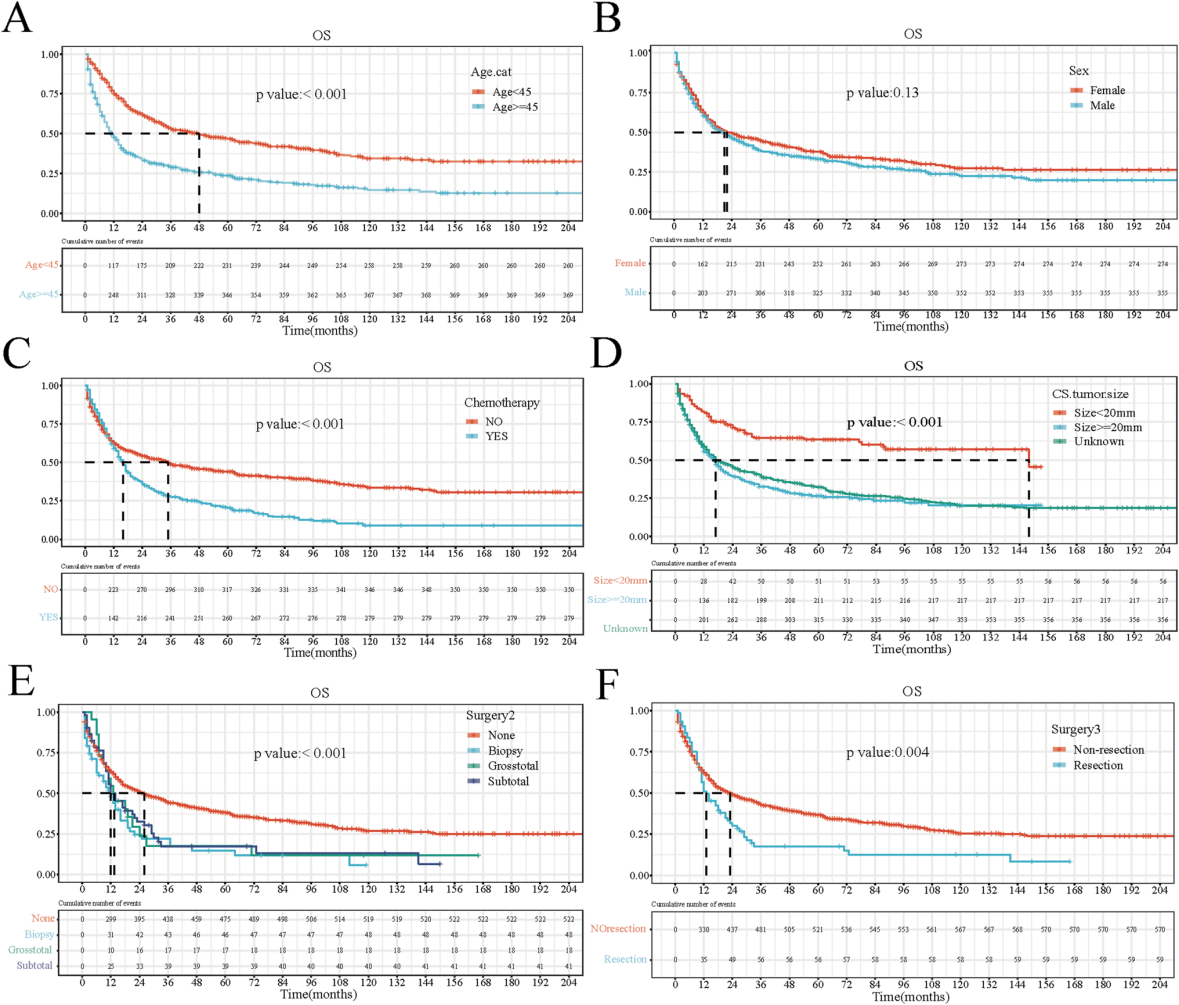
Kaplan-Meier curves for overall survival (OS). **A** Age (<45 years or ≥45 years); **B** gender (male or female); **C** chemotherapy or not; **D** tumor size < 20 mm, ≥20 mm, or unknown; **E** surgery (no operation, biopsy, subtotal resection, or total resection); **F** surgical method (resection or non-resection)

From the KM survival curve of OS (Fig. 1), according to the median survival time, the prognosis of age < 45 years (48 months) is better than that of age ≥ 45 years (12 months) (*P* = 0.00); no chemotherapy (35 months) was better than chemotherapy patients (16 months) (*P* = 0.00). Surgically, non-resection (23 months) was significantly better than resection (13 months) (*P* = 0.00). Similarly, the KM curve of DSS is shown in Fig. A.1.

### Cumulative risk curve for each variable

**Fig. 2:**
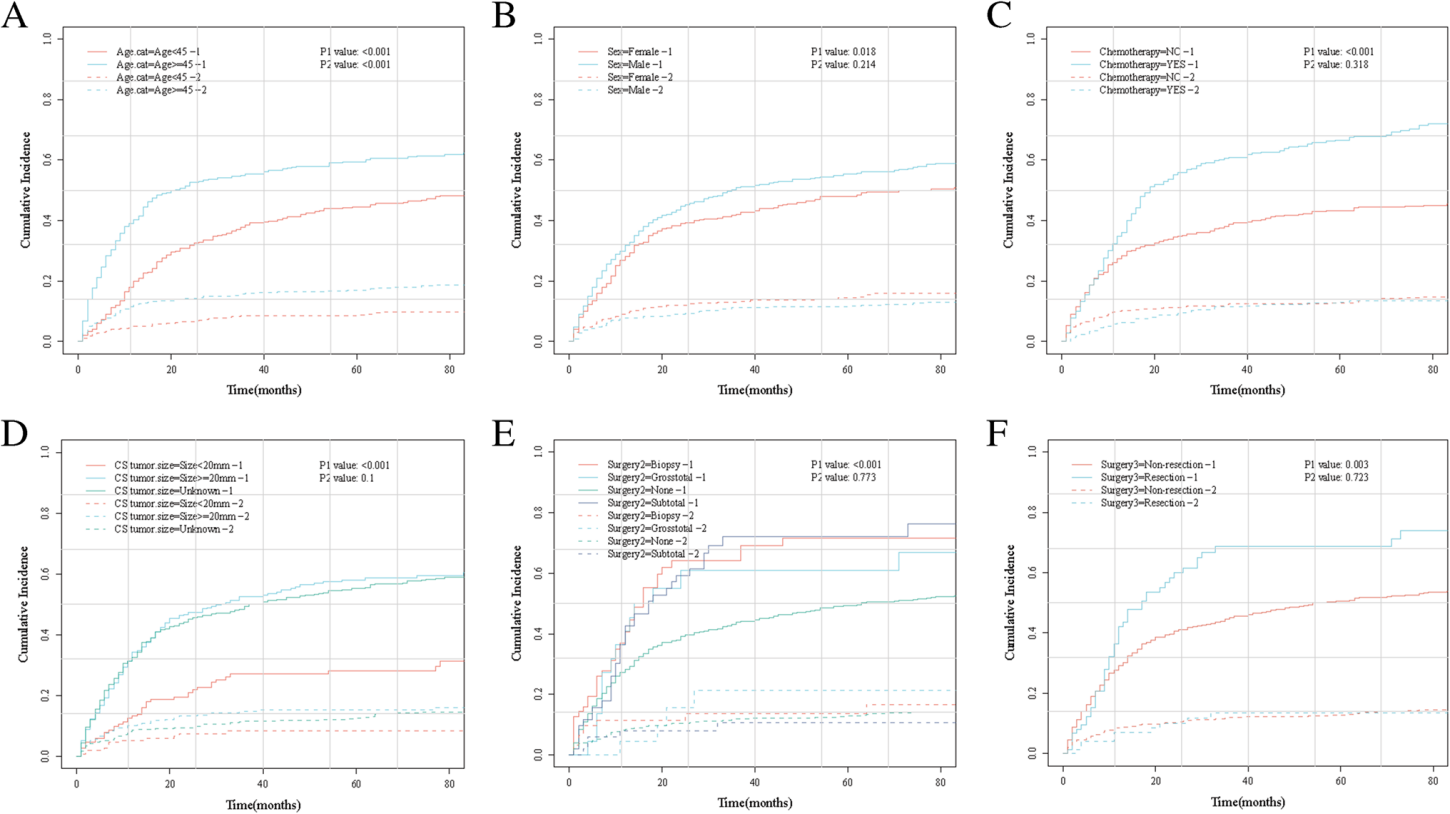
Cumulative risk curve of each variable. **A** Age (<45 years or ≥45 years); **B** gender (male or female); **C** chemotherapy or not; **D** tumor size < 20 mm, ≥20 mm, or unknown; **E** surgical methods (no operation, biopsy, subtotal resection, and total resection); **F** surgical methods (resection or non-resection)

Among the 973 cases, 500 (51.4%) died of glioma and 129 (13.3%) died of other events. The cumulative risk of death from other events was statistically significant between age < 45 and ≥45 years (*P* = 0.00). There was no statistically significant difference across gender (*P* = 0.21), chemotherapy (*P* = 0.32), tumor size (*P* = 0.1), and resection (*P* = 0.72). However, the cumulative risk of death due to glioma was statistically significant across age (*P* = 0.00), gender (*P* = 0.02), chemotherapy (*P* = 0.00), tumor size (*P* = 0.00), surgical methods (*P* = 0.00), and resection (*P* = 0.00) (Fig. 2).

### Screening factors affecting prognosis based on competitive risk model

Due to the impact of non-glioma deaths events, the Cox survival analysis (Table A.1.) would overestimate the incidence of events, so we introduced a competing risk model; multivariate analysis found age ≥ 45 years (HR = 1.57, 95% CI [1.31–1.90], *P* = 0.00), chemotherapeutic (HR = 1.48, 95% CI [1.2–1.83], *P* = 0.00), and tumor size ≥ 20 mm (HR = 1.81, 95% CI [1.29–2.53], *P* = 0.00) to still be unfavorable factors. Absence of a previous history of glioma (HR = 0.48, 95% CI [0.37–0.61], *P* = 0.00) was a favorable prognostic factor in adult brainstem HGG (Table 2).

**Table 2:** Single-factor and multi-factor analyses based on competing risk model

Characteristics	*n* (%)	Univariate analysis		Multivariate analysis	
		SHR (95% CI)	*P* value	SHR (95% CI)	*P* value
**Age (ref = age < 45)**					
Age ≥ 45	488 (50.2)	1.647 (1.386–1.958)	<0.001	1.573 (1.305–1.895)	<0.001
**Sex (ref = female)**					
Male	525 (54)	1.238 (1.039–1.475)	0.017	1.157 (0.964–1.388)	0.12
**Race (ref = White)**					
Black	104 (10.7)	0.88 (0.66–1.175)	0.39	0.967 (0.709–1.319)	0.83
Others	102 (10.5)	0.815 (0.606–1.096)	0.18	0.778 (0.573–1.056)	0.11
**Marital (ref = married)**					
Divorced/separated	89 (9.1)	0.962 (0.696–1.329)	0.81	1.073 (0.767–1.502)	0.68
Single/unmarried	291 (29.9)	0.815 (0.669–0.993)	0.043	1.126 (0.909–1.395)	0.28
Widowed/others	88 (9)	0.905 (0.645–1.27)	0.56	1.075 (0.756–1.529)	0.69
**Diagnosis (ref = 1998~2004)**					
2005~2009	255 (26.2)	0.954 (0.767–1.188)	0.68	0.996 (0.771–1.288)	0.98
2010~2012	184 (18.9)	0.833 (0.646–1.074)	0.16	0.845 (0.621–1.149)	0.28
2013~2016	303 (31.1)	0.631 (0.49–0.812)	<0.001	0.675 (0.498–0.916)	0.011
**Past history type (ref = GBM)**					
Others	793 (81.5)	0.369 (0.302–0.451)	<0.001	0.476 (0.372–0.609)	<0.001
**Radiotherapy (ref = no)**					
Yes	109 (11.2)	1.864 (1.498–2.32)	<0.001	1.145 (0.712–1.843)	0.58
**Chemotherapy (ref = no)**					
Yes	359 (36.9)	1.793 (1.51–2.13)	<0.001	1.484 (1.2–1.834)	<0.001
**Tumor size (ref = size < 20 mm)**					
Size ≥ 20 mm	310 (31.9)	2.389 (1.725–3.308)	<0.001	1.806 (1.291–2.526)	<0.001
Unknown	512 (52.6)	2.362 (1.729–3.226)	<0.001	1.96 (1.4–2.742)	<0.001
**Surgery 2 (ref = none)**					
Biopsy	63 (6.5)	1.685 (1.221–2.325)	0.002	1.012 (0.602–1.7)	0.96
Gross total	22 (2.3)	1.413 (0.843–2.37)	0.19	0.698 (0.367–1.328)	0.27
Subtotal	52 (5.3)	1.701 (1.257–2.302)	0.001	1.029 (0.617–1.716)	0.91

*SHR*, subdistribution hazard ratio

### Based on the competitive risk model, the factors that affect the prognosis after PSM

After PSM, 155 people were included in the analysis, of which 89 underwent non-resection and 66 underwent resection. Match results are unbiased (Table A.2, Fig. A.2). Based on the competing risk model, results showed that absence of a previous history of glioma (HR = 0.35, 95% CI [0.22–0.56], *P* = 0.00) was the only favorable factor for brainstem HGG in adults. For others, such as age (HR = 1.51, 95% CI [0.99–2.34], *P* = 0.07), radiotherapy (HR = 1.05, 95% CI [0.59–1.86], *P* = 0.87), and tumor size (HR = 1.13, 95% CI [0.53–2.37], *P* = 0.75), the prognosis of adult brainstem HGG was not statistically different. It would be worth noting that resection (HR = 0.79, 95% CI [0.53–1.19], *P* = 0.26) was a favorable factor (HR < 1.0), although the results are still not statistically different (Table 3).

**Table 3:** Based on the competing risk model, after propensity score matching, results of single-factor and multi-factor analyses regarding resection

Characteristics	*n* (%)	Univariate analysis		Multivariate analysis	
		SHR (95% CI)	*P* value	SHR (95% CI)	*P* value
**Age (ref = age < 45)**					
Age ≥ 45	74 (47.7)	1.892 (1.283–2.791)	0.001	1.505 (0.968–2.341)	0.069
**Sex (ref = female)**					
Male	84 (54.2)	1.535 (1.013–2.326)	0.043	1.11 (0.628–1.963)	0.72
**Race (ref = Black)**					
Others	18 (11.6)	0.857 (0.364–2.021)	0.72	0.718 (0.235–2.193)	0.56
White	121 (78.1)	1.17 (0.588–2.329)	0.65	0.97 (0.439–2.143)	0.94
**Marital (ref = married)**					
Divorced/separated	9 (5.8)	0.854 (0.396–1.842)	0.69	0.984 (0.392–2.468)	0.97
Single/unmarried	38 (24.5)	0.785 (0.497–1.239)	0.3	1.125 (0.668–1.895)	0.66
Widowed/others	6 (3.9)	0.938 (0.262–3.36)	0.92	0.879 (0.244–3.161)	0.84
**Diagnosis (ref = 1998~2004)**					
2005~2009	39 (25.2)	0.8 (0.491–1.303)	0.37	0.873 (0.469–1.625)	0.67
2010~2012	28 (18.1)	0.861 (0.506–1.464)	0.58	1.031 (0.466–2.281)	0.94
2013~2016	46 (29.7)	0.609 (0.338–1.098)	0.099	0.686 (0.292–1.607)	0.39
**Past history type (ref = GBM)**					
Others	81 (52.3)	0.336 (0.228–0.497)	<0.001	0.347 (0.215–0.561)	<0.001
**Radiotherapy (ref = no)**					
Yes	98 (63.2)	1.407 (0.891–2.223)	0.14	1.049 (0.592–1.861)	0.87
**Chemotherapy (ref = no)**					
Yes	72 (46.5)	1.235 (0.831–1.836)	0.3	0.877 (0.475–1.618)	0.67
**Tumor size (ref = size < 20 mm)**					
Size ≥ 20 mm	63 (40.6)	1.474 (0.773–2.812)	0.24	1.126 (0.534–2.372)	0.75
Unknown	84 (54.2)	1.375 (0.734–2.577)	0.32	1.115 (0.516–2.411)	0.78
**Surgery 3 (ref = non-resection)**					
Resection	66 (42.6)	0.966 (0.657–1.419)	0.86	0.791 (0.527–1.188)	0.26

*SHR*, subdistribution hazard ratio

### CS of resection after PSM

**Fig. 3:**
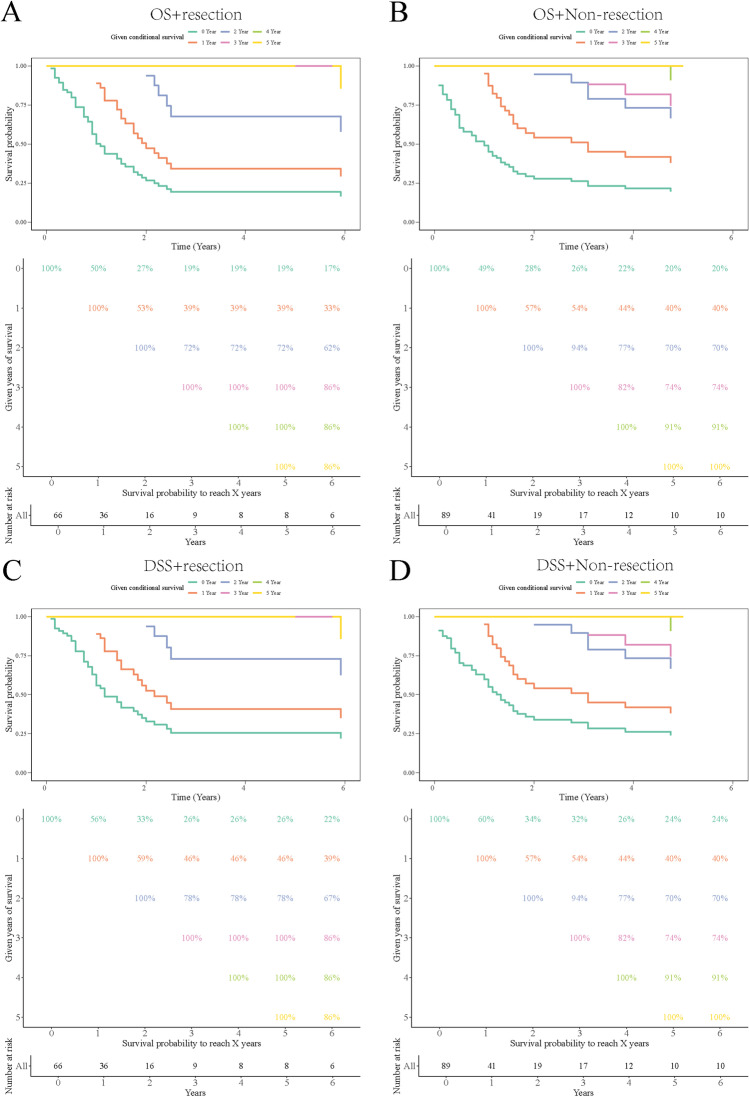
The CS of resection and non-resection after PSM. **A** Overall conditional survival rate after resection; **B** overall conditional survival rate after non-resection; **C** disease-specific conditional survival rate after resection; **D** disease-specific conditional survival rate after non-resection. PSM, propensity score matching; CS, conditional survival

Based on conditional survival, after resection of adult brainstem HGG, patient survival rate remained stable during 3–5 years (Fig. 3A, C). In terms of OS, after 1 year of resection, the survival rate for 3–5 years was 39%; after 2 years of survival, the survival rate for 3–5 years was 72%. After 3 years of survival, the survival rate was 100% at 4–5 years (Fig. 3A). Also in terms of DSS, after 1 year of resection, the survival rate during the 3–5 years was 46%; after 2 years of survival, the survival rate during the 3–5 years was 78%. After 3 years of survival, the survival rate at 4–5 years was 100% (Fig. 3C). However, after non-resection, the survival rate decreased year by year (Fig. 3B, D). In particular, after 3 years of survival, the DSS in the fourth and fifth years were 82% and 74%, respectively (Fig. 3D); both were lower than the DSS in the same period after resection (100%, 100%) (Fig. 3C).

## Discussion

### Background on the treatment of adult brainstem HGG

Adult brainstem HGG are highly malignant and associated with a low survival rate; they have always been regarded as the most difficult to treat [16]. They mostly manifest as dizziness, nausea, and vomiting, along with symptoms such as limb numbness, facial paralysis, and dysphagia [17–19]. With the development of neuronavigation [20, 21], electrophysiological monitoring and diffusion-weighted magnetic resonance imaging technology [22], convection-enhanced separation and application of gamma knife and cyberknife [23–25], histological diagnosis [26], continuous upgradation of radiotherapy, and that of anti-angiogenesis, monoclonal antibodies, and other chemotherapeutic drugs [27, 28], the treatment of adult brainstem HGG has become more feasible. However, the overall survival of patients with adult brainstem HGG and related survival factors are still unclear. Our current study revealed the following: first, age, tumor size, and previous history of glioma are closely related to adult brainstem HGG. Second, in terms of treatment measures, there is no significant statistical difference in improving the overall survival rate of patients with radiotherapy and chemotherapy. Third, and most importantly, for adults with brainstem HGG resection, patients have a significant survival benefit after 3 years of survival.

### The impact of competing events on traditional survival analysis

If there are more than 10% competing events, such as heart disease and car accidents in cancer patients, the use of Cox survival analysis may lead to an incorrect estimation. In this data, we found 20.51% (129/629) of the deaths to be due to non-HGG causes. Therefore, use of a competing risk model would facilitate the accurate assessment of the association between the surgical approach and the prognosis of HGG; multivariate analysis found age ≥ 45 years, chemotherapy, and tumor diameter ≥ 20 mm to be poor prognostic factors. This was basically consistent with the results of Dey et al. [29]. At the same time, compared with low-grade glioma, the more aggressive the tumor, the worse the prognosis of patients with brainstem glioma [30, 31]. Furthermore, factors associated with improved 5-year overall survival included female gender, higher income, and fewer comorbidities [32].

### Effects of radiotherapy on adult brainstem HGG

Radiotherapy is one of the main treatment methods for tumors. However, studies have reported that radiotherapy is significantly related to an increased risk of secondary malignant tumors [33]. For sub-ependymal tumors, radiotherapy had no statistically significant impact on overall survival [34]. Nevertheless, a study by Reithmeier et al. [35] suggested that postoperative radiotherapy is the cornerstone of treatment and can reduce the risk of death by 0.4 times. Compared with radiotherapy alone, postoperative radiotherapy combined with chemotherapy has a significant survival benefit for HGG patients with WHO grade IV (HR: 0.46, 95% CI [0.28–0.76], *P* = 0.00) [36]. For HGG of the pons, radiotherapy remains the standard treatment, although it only provides a survival advantage of 3 to 4 months [37]. But, our study showed that radiotherapy (HR = 1.05, 95% CI [0.59–1.86], *P* = 0.87) had no statistically significant effect on the prognosis of adult brainstem HGG. Research by Hu et al. [38] also confirmed the same.

### Effect of resection on adult brainstem HGG

Whether an adult brainstem HGG would need surgery and the scope of surgical resection have remained controversial. Our study found that resection of adult brainstem HGG has a higher risk of early death, although a stable period is reached after 3 years of survival. In a regression analysis conducted by Majchrzak et al. [39] on 47 adult patients who had undergone surgery for brainstem glioma, the average progression-free survival time for malignant brainstem glioma was 14 months. While the average survival time was 20 months, partial resection of diffuse brainstem glioma did not extend the average survival time by more than 5 years. Rigamonti et al. [40] believed that tumor grade is the only factor that has a statistically significant impact on survival time (*P* = 0.00), whereas younger age, better physical status, and resection surgery showed prolonged survival. However, owing to the special brainstem site, surgery itself is highly risky, and the incidence of complications, such as postoperative bleeding, infection, edema, cerebral hernia, and respiratory insufficiency, is high (36.4%) [35, 41]. If there is a clear boundary between the tumor, normal cerebellum, and tentorium in imaging results, the tumor can be biopsied, and its exogenous part, the brainstem surface, and the accessible part can be roughly/completely removed. Total resection is not possible, since high-grade tumors have strong invasiveness [42].

### Limitations and strengths

First, it is a retrospective study with inherent limitations. With modern medical technology development, the survival rate of patients with adult brainstem HGG will also change. Secondly, the SEER database lacked detailed information about the patient’s physical condition, such as family history and specific parts of tumor (midbrain, pons, or medulla oblongata), especially performance status (PS), is often positively correlated with the prognosis of patients. Thirdly, the database still lacks data of early postoperative mortality, which is of great significance in evaluating the safety of resection. Nevertheless, considering the huge sample size provided by the SEER database, the statistical results would still be very meaningful. In addition, the CS could be incorporated as a factor of survival time, which could assess the prognosis in survivors with HGG more accurately over time.

## Conclusion

The purpose of this study was to investigate the prognostic value of brainstem HGG resection in adults using competing risk models, PSM, and CS based on the SEER database. The data show that the DSS of adult brainstem HGG is 82% and 74% in 1–2 years after non-resection survival for 3 years, but there is a stable survival rate (100%) in 1–2 years after 3 years of resection survival, which will be more effective in helping young patients cope with future uncertainty. Therefore, these data support neurosurgeons to perform maximal safe resection of adult brainstem HGG when available.

## Supplementary Information

Fig. A.1. Kaplan-Meier curves for disease-specific survival (DSS). A: Age (< 45 years or ≥ 45 years); B: gender (male or female); C: chemotherapy or not; D: tumor size < 20 mm, ≥ 20 mm, or unknown; E: surgery (no operation, biopsy, subtotal resection, and total resection); F: surgical method (resection or non-resection). (PNG 137 kb)

High Resolution Image (TIF 14791 kb)

Fig. A.2. PSM and assessment based on whether or not resection was performed. A, C: Before propensity matching; B, D: after propensity matching; E: love plot (PNG 42 kb)

High Resolution Image (TIF 10860 kb)

Table A.1 (DOCX 22 kb)

Table A.2 (DOCX 20 kb)
