# Supplementary figures and images for: Exploration of the prognostic value of the resection of adult brainstem high-grade glioma based on competing risk model, propensity score matching, and conditional survival rate
Source: Neurol Sci. 2023 Jan 6;44(5):1755–64. doi: 10.1007/s10072-022-06557-z (PMC10102064; doi:10.1007/s10072-022-06557-z)

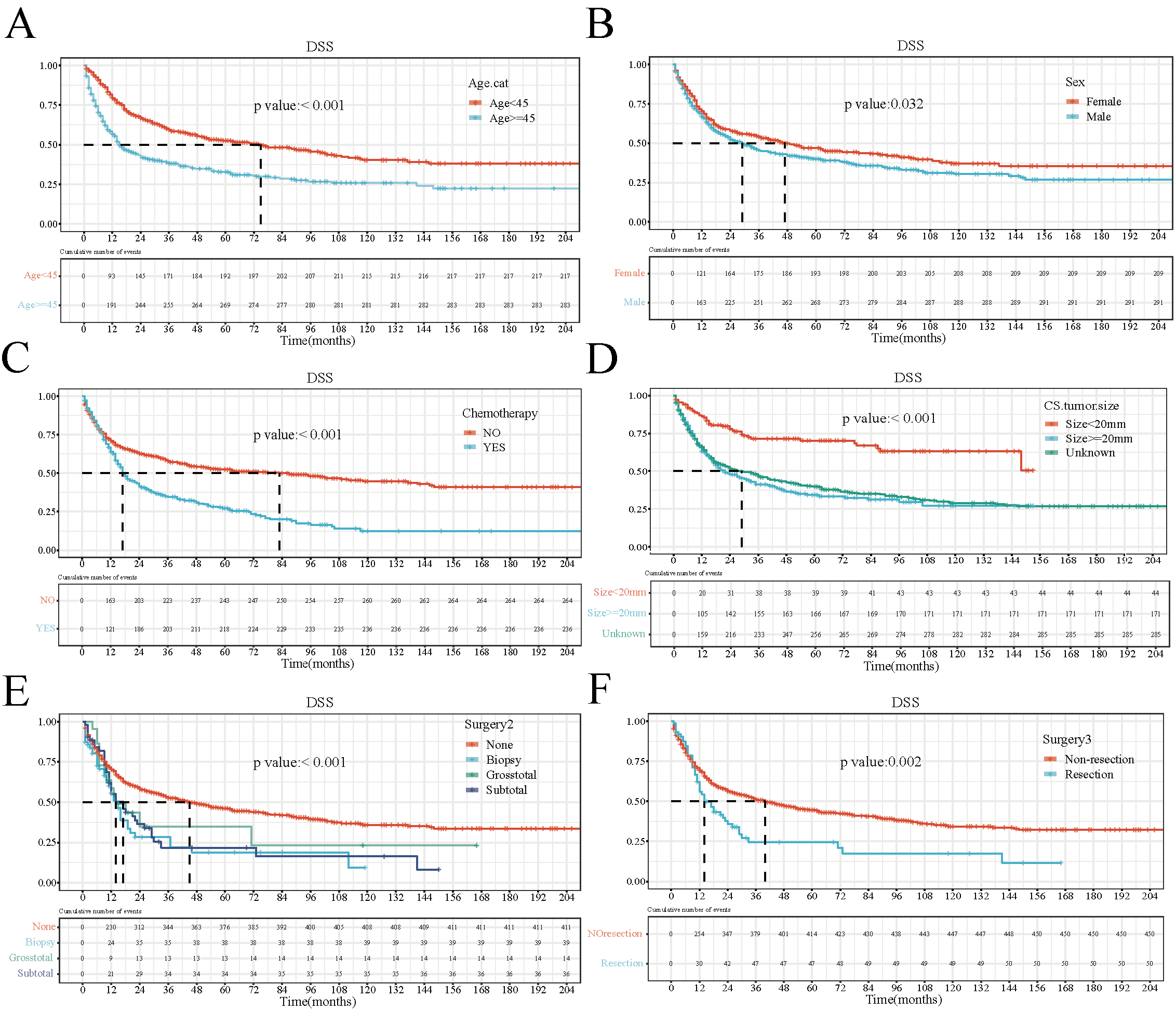

Supplement: Supplementary file 1 — Kaplan-Meier curves for disease-specific survival (DSS). A: Age (< 45 years or ≥ 45 years); B: gender (male or female); C: chemotherapy or not; D: tumor size < 20 mm, ≥ 20 mm, or unknown; E: surgery (no operation, biopsy, subtotal resection, and total resection); F: surgical method (resection or non-resection). (PNG 137 kb) [file 10072_2022_6557_Fig4_ESM.png]

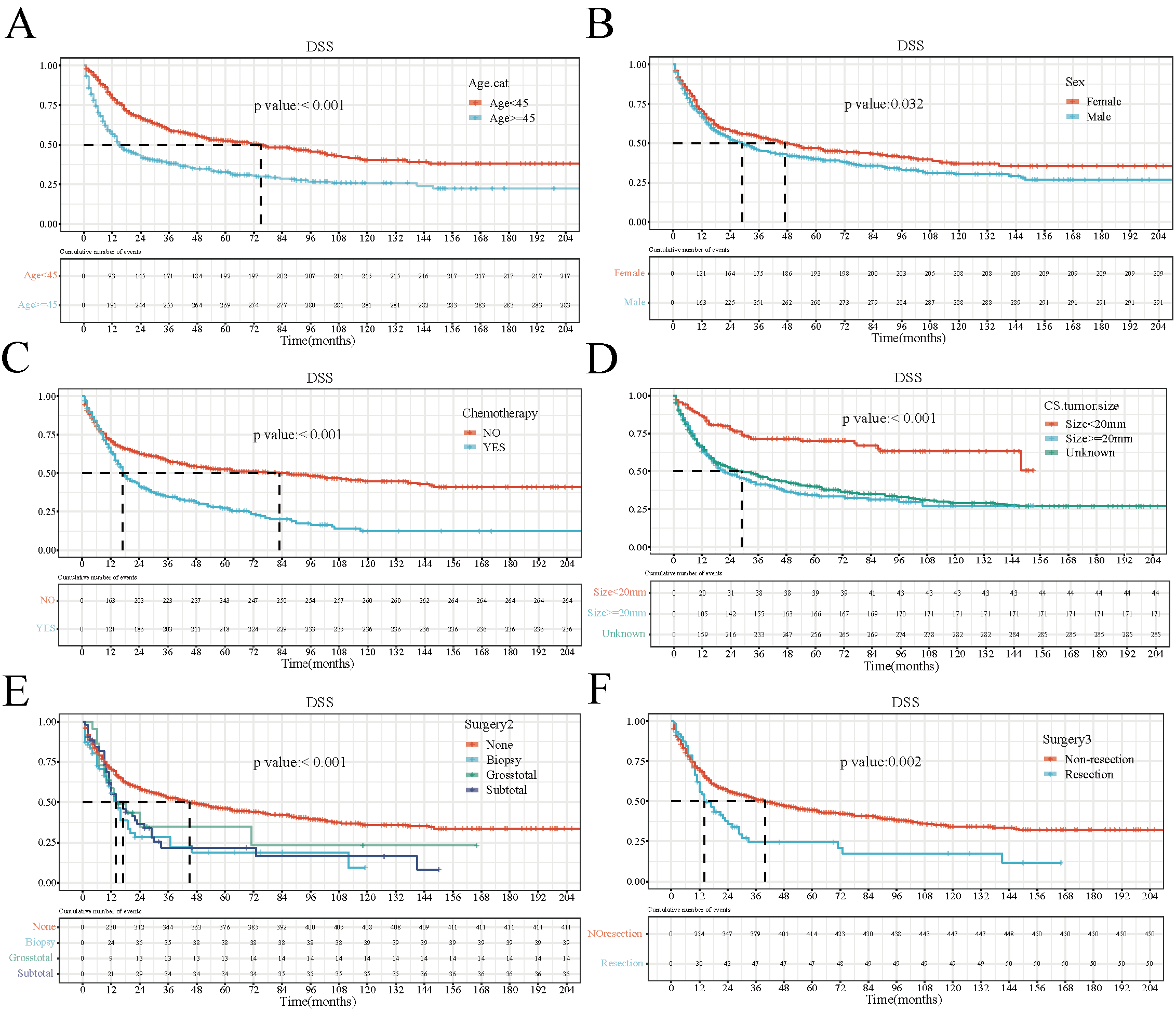

Supplement: Supplementary file 2 — High Resolution Image (TIF 14791 kb) [file 10072_2022_6557_MOESM2_ESM.tif]

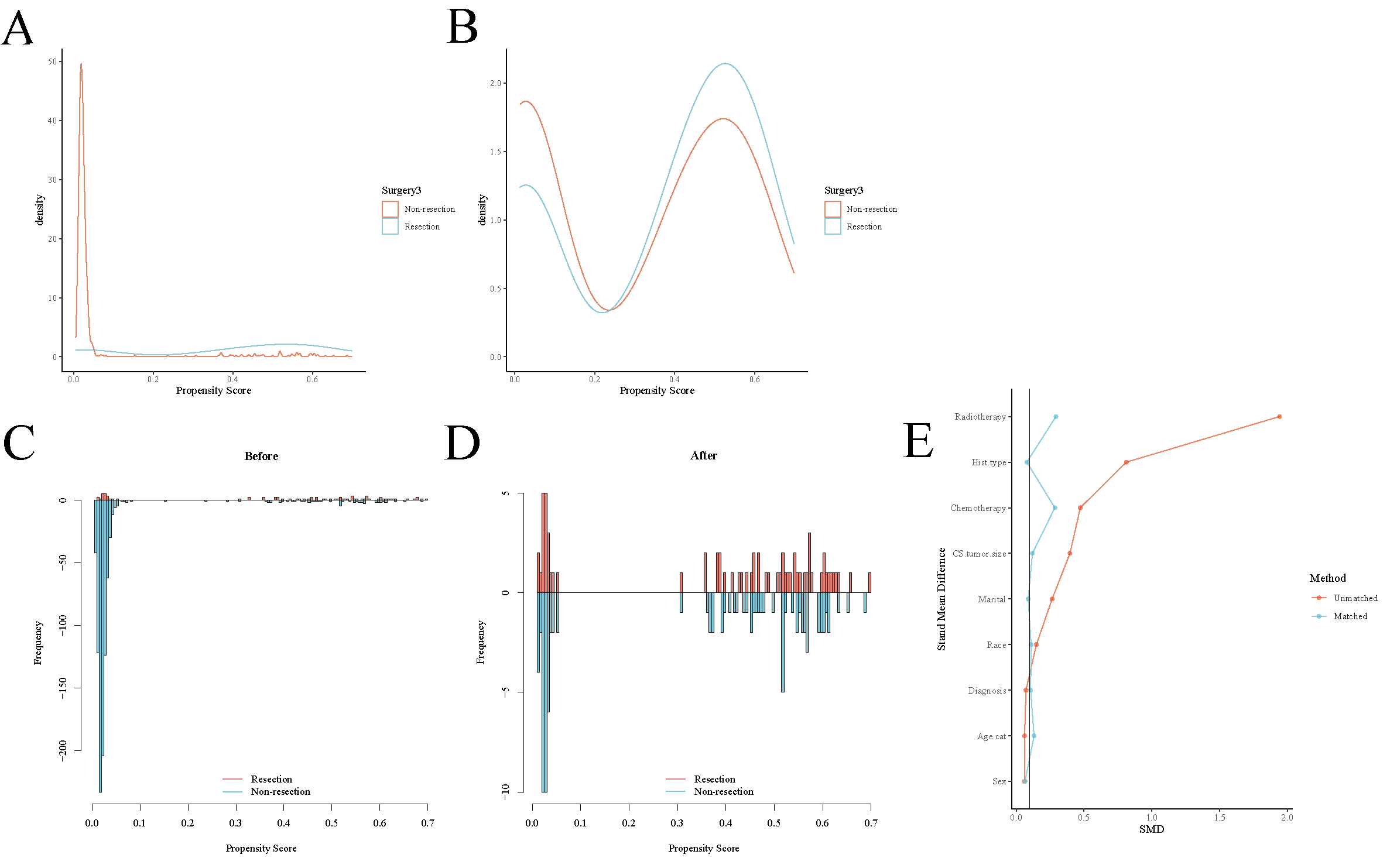

Supplement: Supplementary file 3 — PSM and assessment based on whether or not resection was performed. A, C: Before propensity matching; B, D: after propensity matching; E: love plot (PNG 42 kb) [file 10072_2022_6557_Fig5_ESM.png]

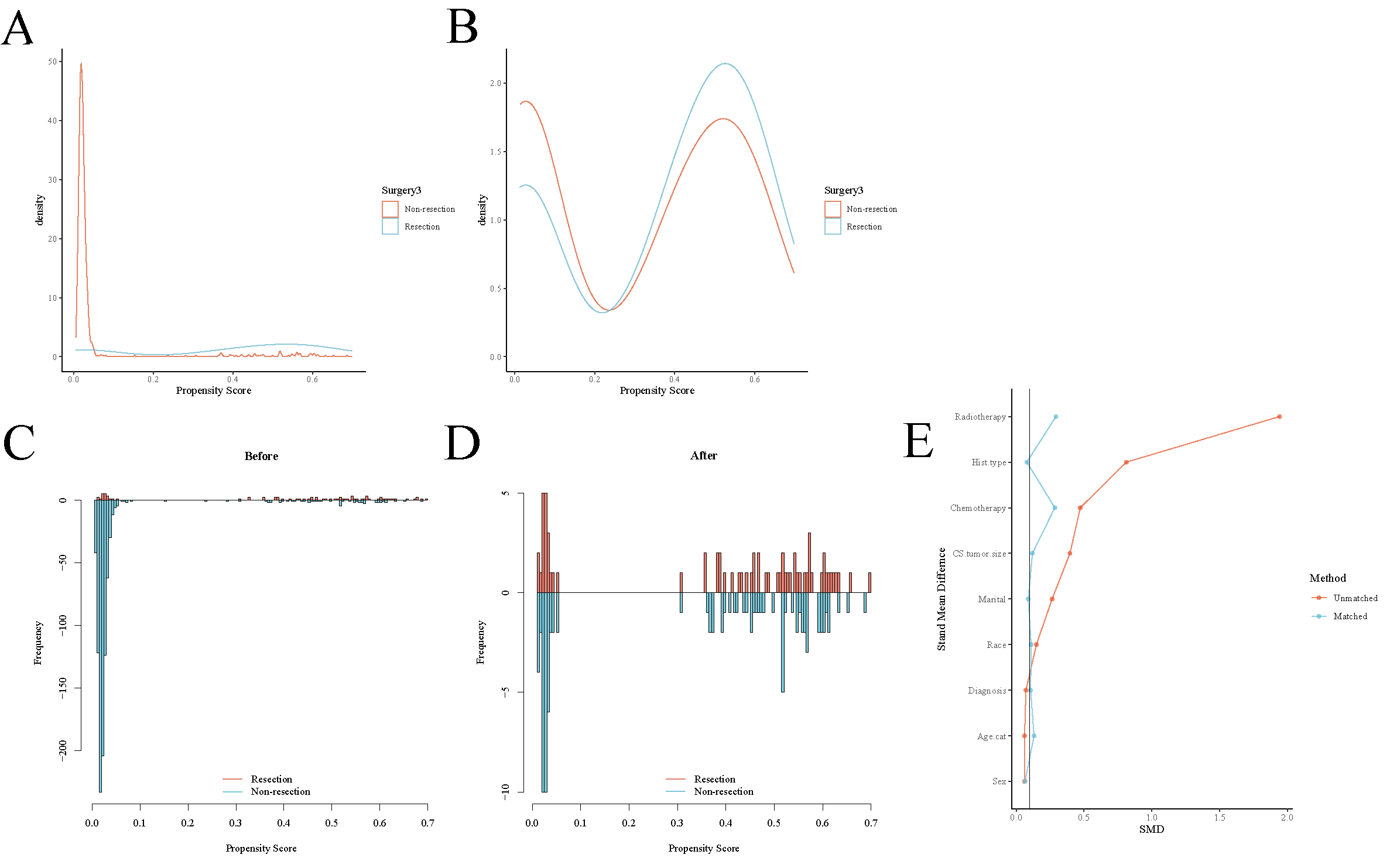

Supplement: Supplementary file 4 — High Resolution Image (TIF 10860 kb) [file 10072_2022_6557_MOESM4_ESM.tif]
