# Supplementary material for: Exploration of the prognostic value of the resection of adult brainstem high-grade glioma based on competing risk model, propensity score matching, and conditional survival rate
Source: Neurol Sci. 2023 Jan 6;44(5):1755–64. doi: 10.1007/s10072-022-06557-z (PMC10102064; doi:10.1007/s10072-022-06557-z)
Supplement: Supplementary file 5 — (DOCX 22 kb) [file 10072_2022_6557_MOESM5_ESM.docx]

Table A.1.

Univariate and multivariate Cox regression analyses of prognostic factors of adult brainstem HGG

|  |  | Univariate analysis | | Multivariate analysis | |
| --- | --- | --- | --- | --- | --- |
| Charateristics | n (%) | HR (95% CI) | P value | HR (95% CI) | P value |
| **Age:（ ref = Age < 45）** | | | | | |
| Age >= 45 | 488(50.2) | 2.121(1.808-2.488) | < 0.001 | 2.063(1.73-2.459) | < 0.001 |
| **Sex: （ref = Female）** |  |  |  |  |  |
| Male | 525(54) | 1.128(0.964-1.321) | 0.134 | 1.044(0.889-1.226) | 0.6 |
| **Race: （ref = White）** |  |  |  |  |  |
| Black | 104(10.7) | 0.897(0.693-1.162) | 0.41 | 0.946(0.723-1.237) | 0.685 |
| Others | 102(10.5) | 0.788(0.597-1.041) | 0.094 | 0.691(0.52-0.917) | 0.011 |
| **Marital:（ref = Married）** | | | | | |
| Divorced/Separated | 89(9.1) | 1.145(0.875-1.498) | 0.324 | 1.183(0.897-1.561) | 0.234 |
| Single/Unmarried | 291(29.9) | 0.792(0.659-0.953) | 0.013 | 1.207(0.988-1.474) | 0.065 |
| Widowed/Others | 88(9) | 0.966(0.73-1.279) | 0.81 | 1.114(0.836-1.485) | 0.462 |
| **Diagnosis:（ref = 1998~2004）** | | | | | |
| 2005~2009 | 255(26.2) | 0.822(0.67-1.008) | 0.059 | 0.832(0.658-1.054) | 0.128 |
| 2010~2012 | 184(18.9) | 0.813(0.646-1.023) | 0.077 | 0.811(0.619-1.063) | 0.129 |
| 2013~2016 | 303(31.1) | 0.704(0.562-0.882) | 0.002 | 0.75(0.574-0.98) | 0.035 |
| **Past history type: （ref = GBM）** | | | | | |
| Others | 793(81.5) | 0.35(0.291-0.421) | < 0.001 | 0.457(0.369-0.565) | < 0.001 |
| **Radiotherapy: （ref = No）** | | | | | |
| Yes | 109(11.2) | 1.673(1.332-2.102) | < 0.001 | 0.736(0.489-1.108) | 0.142 |
| **Chemotherapy: （ref = No）** | | | | | |
| Yes | 359(36.9) | 1.624(1.385-1.905) | < 0.001 | 1.393(1.156-1.678) | < 0.001 |
| **tumor size: （ref = Size < 20mm）** | | | | | |
| Size>=20mm | 310(31.9) | 2.738(2.039-3.676) | < 0.001 | 2.064(1.521-2.801) | < 0.001 |
| Unknown | 512(52.6) | 2.491(1.878-3.302) | < 0.001 | 2.031(1.492-2.764) | < 0.001 |
| **Surgery2: （ref = None）** | | | | | |
| Biopsy | 63(6.5) | 1.888(1.403-2.541) | < 0.001 | 1.65(1.065-2.557) | 0.025 |
| Grosstotal | 22(2.3) | 1.49(0.931-2.385) | 0.097 | 0.96(0.549-1.68) | 0.887 |
| Subtotal | 52(5.3) | 1.566(1.139-2.155) | 0.006 | 1.257(0.803-1.969) | 0.318 |

Surgery 2：Four categorical variables（0：None；1：Biopsy；2：subtotal resection；3：gross total resection）
