# Supplementary material for: Exploration of the prognostic value of the resection of adult brainstem high-grade glioma based on competing risk model, propensity score matching, and conditional survival rate
Source: Neurol Sci. 2023 Jan 6;44(5):1755–64. doi: 10.1007/s10072-022-06557-z (PMC10102064; doi:10.1007/s10072-022-06557-z)
Supplement: Supplementary file 6 — (DOCX 20 kb) [file 10072_2022_6557_MOESM6_ESM.docx]

| Table A.2.  Baseline table of each variable after propensity score matching | | | | | | |
| --- | --- | --- | --- | --- | --- | --- |
| Matched | level | Overall | Non-resection | Resection | p | SMD |
| n |  | 155 | 89 | 66 |  |  |
| Age (%) | Age < 45 | 81 (52.3) | 49 (55.1) | 32 (48.5) | 0.517 | 0.132 |
|  | Age ≥ 45 | 74 (47.7) | 40 (44.9) | 34 (51.5) |  |  |
| Sex (%) | Female | 71 (45.8) | 42 (47.2) | 29 (43.9) | 0.811 | 0.065 |
|  | Male | 84 (54.2) | 47 (52.8) | 37 (56.1) |  |  |
| Race (%) | White | 121 (78.1) | 71 (79.8) | 50 (75.8) | 0.793 | 0.11 |
|  | Black | 16 (10.3) | 8 ( 9.0) | 8 (12.1) |  |  |
|  | Others | 18 (11.6) | 10 (11.2) | 8 (12.1) |  |  |
| Marital (%) | Married | 102 (65.8) | 60 (67.4) | 42 (63.6) | 0.96 | 0.089 |
|  | Divorced/Separated | 9 (5.8) | 5 (5.6) | 4 (6.1) |  |  |
|  | Single/Unmarried | 38 (24.5) | 21 (23.6) | 17 (25.8) |  |  |
|  | Widowed/Others | 6 (3.9) | 3 (3.4) | 3 (4.5) |  |  |
| Diagnosis (%) | 1998–2004 | 42 (27.1) | 24 (27.0) | 18 (27.3) | 0.934 | 0.107 |
|  | 2005–2009 | 39 (25.2) | 21 (23.6) | 18 (27.3) |  |  |
|  | 2010–2012 | 28 (18.1) | 16 (18.0) | 12 (18.2) |  |  |
|  | 2013–2016 | 46 (29.7) | 28 (31.5) | 18 (27.3) |  |  |
| Past history type (%) | GBM | 74 (47.7) | 41 (46.1) | 33 (50.0) | 0.747 | 0.079 |
|  | Others | 81 (52.3) | 48 (53.9) | 33 (50.0) |  |  |
| Radiotherapy (%) | No | 57 (36.8) | 38 (42.7) | 19 (28.8) | 0.108 | 0.293 |
|  | Yes | 98 (63.2) | 51 (57.3) | 47 (71.2) |  |  |
| Chemotherapy (%) | No | 83 (53.5) | 53 (59.6) | 30 (45.5) | 0.115 | 0.285 |
|  | Yes | 72 (46.5) | 36 (40.4) | 36 (54.5) |  |  |
| tumor size (%) | Size < 20 mm | 8 (5.2) | 5 (5.6) | 3 (4.5) | 0.762 | 0.12 |
|  | Size ≥ 20 mm | 63 (40.6) | 34 (38.2) | 29 (43.9) |  |  |
|  | Unknown | 84 (54.2) | 50 (56.2) | 34 (51.5) |  |  |
